# Health-seeking behaviour in the city of Lubumbashi, Democratic Republic of the Congo: results from a cross-sectional household survey

**DOI:** 10.1186/1472-6963-14-173

**Published:** 2014-04-15

**Authors:** Mukalenge F Chenge, Jean Van der Vennet, Numbi O Luboya, Veerle Vanlerberghe, Mala A Mapatano, Bart Criel

**Affiliations:** 1 Ecole de santé publique, Université de Lubumbashi, P.O. Box 1825, Lubumbashi, RD, Congo; 2 Department of Public Health, Institute of Tropical Medicine, Nationalestraat 155, 2000 Antwerp, Belgium; 3 Ecole de Santé Publique, Université de Kinshasa, P.O. Box 11850, Kinshasa I, RD, Congo

**Keywords:** Democratic Republic of the Congo, Lubumbashi, Urban health, Health care system, Health-seeking behaviour, Health service utilization, Health care costs

## Abstract

**Background:**

Concerns about the occurrence of disease among household members generally initiate treatment-seeking actions. This study aims to identify the various treatment-seeking options of patients in Lubumbashi, analyze their health-seeking behaviour, identify determinants for the use of formal care, and analyze direct health care expenditure.

**Methods:**

A cross-sectional survey of households in Lubumbashi was conducted in July 2010. Information was collected from a randomly selected sample of 251 households with at least one member who had been ill in the 2 weeks preceding the survey.

**Results:**

Frequently used initial treatment-seeking options consist of self-medication based on modern medicines (54.6%), the use of first-line health services (23.1%) and hospitals (11.9%), with a perceived effectiveness of 51%, 83% and 91% respectively. If people go for a second option, then formal health care services are most often preferred. The majority (60%) of patients’ spontaneous itineraries reflect the expected functioning of a local health care system, with a patient flow characterised by the use of a first line health facility prior to the use of hospital-based services. Chronicity of the disease is the main determinant of seeking formal care. Analysis of care expenditure reveals that drugs are the only line of expenditure in the informal system and the main source of expenditure in the formal system; costs do not discriminate between first-line health services and hospitals, and the payment system is regressive since the poorest patients pay the same amounts as the richest.

**Conclusions:**

This study points to the importance of self-medication as the first therapeutic option for the majority of patients in Lubumbashi, whatever the nature of the health problem. There is a lot of room to rationalise this practice. Although formal care is not common initial therapeutic option, it is the source of care most patients turn to, especially when they believe having a chronic disease. Patients’ itineraries in this urban environment are complex; health managers should try and deal with this reality. Finally, our study indicates that poor patients face the same level of out-of-pocket payments as the more wealthy ones, hence the need for more equitable health care financing arrangements.

## Background

Concerns about the occurrence of disease among household members generally create a demand for treatment and initiate treatment-seeking actions. Decisions are then taken and therapeutic options are reviewed. There is a vast literature on the nature and determinants of these therapeutic options, including in developing countries. Therapeutic options are categorized into the use of public versus private, private for profit versus private not for profit, formal versus non-formal, modern versus traditional and first-line versus second-line health care services [1-6]. The choice of therapeutic options is determined by demographics, socio-economic concerns, health problems, and the features of the various health services [7].

While studies are published on health seeking behaviour in rural sub-Saharan African settings, very few are devoted to urban environments. Those that exist were published before 2000 [1,8-10], hence the need for more recent data. This scarcity of information limits our understanding of the health-seeking behaviour of patients in urban contexts, i.e. one in which the health care system is characterized, among other things, by a diversity of supply [2,11-14] and by an ongoing epidemiological transition with an increase of non communicable diseases.

The DR Congo (Democratic Republic of the Congo) is currently conducting a health system reform. Given the contextual differences between rural and urban environments, the city of Lubumbashi has been chosen to pilot the reform in Congolese urban settings.

The epidemiological profile of the DR Congo is characterized by endemic diseases like malaria, typhoid fever, tuberculosis, and leprosy. While outbreaks of measles, Ebola hemorrhagic fever, whooping cough, and cholera continue to strike, one observes that non-communicable diseases like diabetes, hypertension, and sickle cell anaemia are on the rise [15]. The ratios of infant and maternal mortality are among the highest in the world, with 97 infant deaths per 1000 live births [16] and 549 maternal deaths per 100,000 live births [17].

This general picture of morbidity and mortality in the DR Congo also prevails in the city of Lubumbashi. In 2002, the Observatory of Urban Change (Observatoire du Changement Urbain) of the University of Lubumbashi published a report on the health profile of the inhabitants of Lubumbashi [18]. According to this report, morbidity is dominated by parasitic and infectious diseases. Malaria tops the disease profile at the community level (67% of disease cases), as well as in health care facilities (over 20% of outpatients and inpatients). It is also the pathology most frequently responsible for fever in pregnant women [19] and anaemia (the most common cause of mortality, especially among children under five) [18,20]. In Lubumbashi, complications during pregnancy are the most common cause of maternal mortality [21]. The prevalence of hypertension in the population is estimated at 13% [22].

Despite the magnitude of health problems in Lubumbashi, and despite the availability and diversity of the formal health care delivery system [14], the utilization of curative care in first-line health services barely reaches 0.4 new cases per inhabitant per year [23]. This relatively low utilisation of curative care in formal health services may be explained by financial constraints which lead patients to opt for the use of informal care providers. Given that no significant form of social health protection arrangement is in place in Lubumbashi, out of pocket payment is the common mode of paying for health care.

This study aims to identify the therapeutic options used by the inhabitants of Lubumbashi, analyze individuals’ therapeutic itineraries, identify the determinants of the use of formal care, and analyze direct health care expenditures. Its purpose is to help urban health authorities to set evidence-based priorities in terms of reorganizing health care delivery in the city. Finally, this study is more comprehensive than previous ones limited to study the formal health care sector [14,23].

## Methods

### Study setting

The study was conducted in the city of Lubumbashi in the south-eastern part of the DR Congo. The size of the population was estimated at 2 million inhabitants in 2010. In 2010, Lubumbashi counted nine Health Districts (HDs), which were divided into 107 unequally distributed Health Areas (HAs). The formal health facilities consisted of nine hospitals of 50 beds or more, more than 20 small-sized hospitals (counting less than 50 beds), and an extensive network of First-Line Health Services (FLHS) [14].

### Study design

A cross-sectional study was conducted through a rapid household survey from the 21^st^ to the 24^th^ July 2010. We carried out semi-structured interviews with the heads of household or any other household member able to answer questions.

### Sampling

Sample size was calculated with the aim to detect a 50% formal health care use among household members who experienced a health problem within the two weeks preceding the survey (accuracy 10%, alpha 5%, power 80%). The sample size was sufficient to detect an association of 2.5 between determinants and the outcome with a power of 85%. We used a two-stage cluster sampling (HA cluster and simple random sampling of households). The sample size was corrected for the design effect by multiplying it by two and by increasing it with 20% to account for inaccuracies and non-respondents. The calculated sample size representing the minimal number of households to be surveyed was 231 corresponding to 33 clusters of seven households. The inclusion criteria in the survey were: a household in which someone had been ill within two weeks preceding the survey and in which there was a person able to answer questions. Houses were revisited once (the next day) when nobody was at home during the survey visit.

In practice, we randomly selected three HAs in each HD with less than 10 HAs, four HAs in HDs with 10 to 14 HAs, and five HAs in HDs with at least 15 HAs. Based on a detailed map of each selected HA, we randomly selected seven households corresponding to the minimum number of households to be surveyed by HA. These seven households were each visited and investigated if they met the inclusion criteria. When the household lacked anyone who had been ill or who was able to respond to the investigator, the investigator was instructed to move to the first household to the right when facing the previous one, and to continue this itinerary until he found a household that met the inclusion criteria. If a person had developed more than one episode of disease during the two weeks preceding the survey, the focus was on the most recent one [4]. If several people were ill in a same household, one household member was chosen at random, drawing a number from a bag, for further investigation.

### Data collection

A semi-structured interview guide was developed in order to list all therapeutic options and possible patient itineraries. By therapeutic options, we mean all actions taken to cope with the disease; by itineraries, we mean their sequence within a given disease episode [4,24]. As with most health problems, the recollection of the details of seeking treatment quickly fades, a recall period of two weeks was therefore considered [1,4,25]. The health problem may have appeared within the timespan of these two weeks, or may have started before but continued during at least part of the 2-week recall period [24].

Information about therapeutic options was classified as follows:

– Traditional medicine (self-medication based on traditional remedies or the use of a traditional healer).

– Self-medication based on modern medicines available at home, bought at the market or in a neighbourhood pharmacy.

– Any occasional use of the services of a self-employed health professional (private doctor or nurse) at his home or at the patient’s home.

– Any use of an established first-line health service (i.e. any public or private facility providing ambulatory curative and preventive care, and able to handle normal deliveries, regardless of what the facility was called: health centre, medical centre, dispensary, etc.).

– Any public or private hospital capable of providing major general surgery and hospitalization, regardless of what it was called: hospital, clinic, referral health centre, etc.

The interviewer also collected information related to variables that could explain the choice of a given therapeutic option. Variables reported by the respondent included the patient’s age and sex, household size, number of parents in the household, education and identity of the main breadwinner, nature of the disease, and acuteness or chronicity of the disease (as reported by interviewee). Other variables were assessed through observation, such as connection to electricity and water, type of pavement of the house, and household wealth (possession of following assets: mobile phone, bicycle, motorcycle, or car). The interviewer also asked for the financial expenditure for the care of the health problem.

The interview guide was designed in French (the official language of the DR Congo) and then translated into Swahili by a person familiar with both languages. The Swahili version of the guide was translated back into French by another person who had perfect mastery of the two languages for a final check of the accuracy of the translation. The French and Swahili versions were used throughout the survey, depending on the language preference of the respondent.

Data collection was conducted by 12 nurses; all were members of the district management teams of the nine HDs of Lubumbashi. These nurses were not involved in providing curative care in the health facilities of their respective HDs. They were trained for the purpose of the survey and the interview guide was pre-tested.

### Data analysis

Two trained persons simultaneously recorded all data in an Epi Info database. Statistical analyses were performed using STATA software IC/11 at a statistical significance level of 5%. Therapeutic options were grouped into two categories: informal care (traditional medicine and self-medication based on modern drugs) and formal care (use of a private health professional at his home or at the patient’s home, use of a first-line health service or a hospital).

Referring to other similar health-seeking behaviour studies [1,4,26], we grouped the notified health problems into three main categories: malaria syndrome, including malaria and unspecified fever; respiratory tract syndrome: i.e. common colds, cough, and acute respiratory infections (bronchitis, pneumonia); and Water, Hygiene and Sanitation (WHS) complex: health problems related to water, hygiene, and sanitation (diarrhoea, gastro-intestinal diseases, abdominal pain, and dermatitis). We added a fourth category “others”, which combined health problems not fitting the three first syndromes: for instance, gynaecological and obstetrical problems, and chronic conditions (e.g., diabetes and hypertension).

By adapting the classification of Petit [27], we grouped the main income-generating activities of the head of household into four categories: inactive; paid on a daily basis (taxi drivers, farmers, informal traders); salaried employees and independent self-employed workers (masons, carpenters, cabinet-makers, joiners, plumbers, designers, and small business owners); and corporate executives and professionals (e.g., lawyers and doctors).

In line with the study environment and with reference to other similar studies [1,27], the socioeconomic status of each household was assessed using an index calculated on the basis of weighted categories (Table 1). The score generated varies from 3 to 36.5 and allowed us to classify households into quintiles of socio-economic welfare [1,16].

**Table 1:** Design of socio-economic status assessment of surveyed households, Lubumbashi, 2010

**Variables**	**Score**
Household size	
1-5 persons	3
6-10 persons	2
≥11 persons	1
Number of parents in the household	
1	1
2	3
Level of education of household head	
Illiterate	0.5
Incomplete primary education	1
Complete primary education	1.5
Incomplete secondary education	2
Complete secondary education	2.5
Incomplete Higher education	3
Complete Higher education	3.5
Main income generating activity of the household head	
Inactive	0.5
Paid on a daily basis	1
Salaried employees and independent workers	2
Corporate executives and professionals	4
Assets ownership of the household^a^	
Cell phone	1
Bicycle	2
Motorcycle	4
Car	6
Characteristics of the habitat^a^	
Connection to electricity	2
Ground pavement	4
Connection to water	4
Total score range	3 – 36.5

^a^The score is 0 when the household does not have the item.

**Figure 1:**
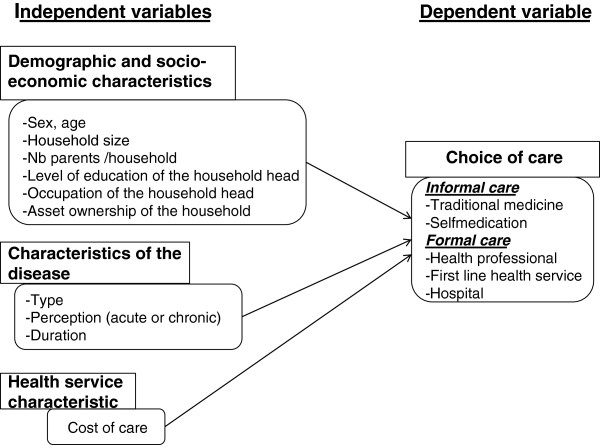
**Factors influencing the choice of care: conceptual framework adapted from Develay et al. **[1]** and Kroeger **[7]**.**

Searching for factors that might influence the initial choice of a therapeutic option in the context of Lubumbashi (Figure 1), we first performed a bivariate analysis with all relevant independent variables. Odds Ratios and their respective 95% Confidence Intervals (CI) were calculated. A binary logistic General Estimating Equation model was constructed from the variables that were significant in the bivariate analysis and these non-significant variables that were deemed important or potential confounders on a priori grounds [28]. This kind of model takes into account the clustering of data [29].

To appreciate the costs of care, we calculated median costs and used 95% CI for comparison purposes.

### Ethical considerations

The study protocol was reviewed and approved by the medical ethical committee of the UNILU (University of Lubumbashi) on 5 July 2010 (Approval No.: UNILU/CEM/009/2010) and the IRB (Institutional Review Board) of the Institute of Tropical Medicine in Antwerp on 7 July 2010 (IRB Number: 10204719). No harm was anticipated to respondents. Interviewers were instructed to explain in advance the objectives of the study and highlight the anonymous and confidential use of collected data. Informed consent of respondents was sought, and signed consent forms were obtained in advance.

## Results

A total of 268 households met the inclusion criteria; one person in each household was interviewed. Seventeen patients (6.3%) did not take any therapeutic action for their health problem. We did not further analyze data from these households. Ultimately, the analysis focused on a sample of 251 households.

### Profile of surveyed households and patients

Table 2 presents the main characteristics defining the profile of households and patients surveyed.

**Table 2:** Key characteristics of households surveyed, Lubumbashi 2010

**Variables**	**n = 251**	**%**
Household size		
1-5	74	29.5
6-10	129	51.4
≥11	48	19.1
Number of parents in the household		
1	6	2.4
2	245	97.6
Level of education of household head		
Illiterate	4	1.6
Primary education	47	18.7
Secondary education	145	57.8
Higher education	55	21.9
Main income generating activity of the household head		
Inactive	29	11.6
Paid on a daily basis	77	30.7
Salaried employees and independent workers	139	55.3
Corporate executives and professionals	6	2.4
Socioeconomic status of the household		
1 (Poorest)	52	20.7
2	60	23.9
3	39	15.5
4	52	20.7
5 (Richest)	48	19.1
Age of patients surveyed (years)		
≤ 4	61	24.3
≥ 5	190	75.7
Sex of patients surveyed		
Male	105	41.8
Female	146	58.2

### Reported health problems

The frequencies of the four groups of health problems reported by respondents are shown in Table 3: of 251 patients, 34.3% (n = 86) of patients experienced a malaria syndrome, 27.9% (n = 70) a respiratory tract syndrome and 17.1% (n = 43) suffered from diseases related to the WHS complex. In addition, 79.4% of respondents in the category “other” believed that the diseases they reported were chronic problems, and 69.8% of patients with a disease duration exceeding 2 weeks were placed in the category “other”, which includes recognized chronic conditions such as diabetes and hypertension.

**Table 3:** Health problems reported by respondents, Lubumbashi 2010

	**Malaria syndrome n = 86**	**Resp. tract syndrome n = 70**	**Complex WHS n = 43**	**Others n = 52**	**Total n = 251**
Acute/chronic disease^a^					
Acute	84 (38.7)^b^	67 (30.9)	41 (18.9)	25 (11.5)	217 (100.0)
Chronic	2 (5.9)	3 (8.8)	2 (5.9)	27 (79.4)	34 (100.0)
Duration					
≤ 2 weeks	80 (38.5)	66 (31.7)	40 (19.2)	22 (10.6)	208 (100.0)
> 2 weeks	6 (14.0)	4 (9.3)	3 (7.0)	30 (69.8)	43 (100.0)

^a^As reported by interviewee.

^b^Figures between () are percentages.

Additionally, by examining responses about acuteness/chronicity of disease, as reported by interviewee, and the actual duration of disease reported, we observed that only 5.9% (2/34) of the diseases that interviewee reported to be chronic lasted for less than 2 weeks and 25.6% (11/43) of conditions that lasted for more than 2 weeks were considered by respondents to be acute problems.

### Therapeutic options and patients’ itineraries

**Figure 2:**
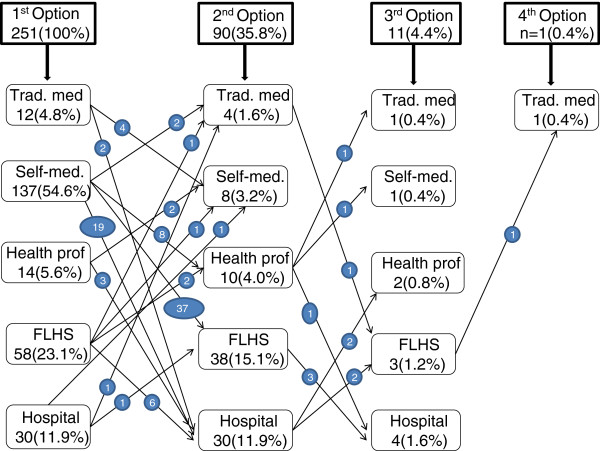
Therapeutic options (boxes) and patient itineraries (arrows) recorded in Lubumbashi, July 2010.

Figure 2 presents the different therapeutic options and describes the various patient itineraries in search of care.

Self-medication based on the use of modern drugs was the initial most widely used option (54.6%), followed respectively by the use of a FLHS (23.1%) and a hospital (11.9%). Traditional medicine as an initial therapeutic option represented less than 5% of patient itineraries.

We also note that 90 of the 251 patients (35.8%) used more than one therapeutic option; patients described as many as 15 different trajectories from their first to their second option. For 80% of these 90 patients, informal care was the initial therapeutic option. The shift from informal care as initial option to formal care as second option was 91.7% (66/72), compared with 33.3% (6/18) in the opposite direction; this difference was statistically significant (p < 0.001). In 60% of cases (54/90), the shift from first to second therapeutic option is consistent with a health-seeking behaviour that health system planners would qualify as “rational” (i.e. starting from self-medication, progressing next to the use of ambulatory care offered by an individual health professional or a first-line health facility, and thereafter the use of a hospital).

Figure 2 also shows that 40.6% of patients surveyed (102/251) used formal health care services as their first therapeutic option. Their main point of entry into the formal system was a FLHS in 56.5% of cases (95/168), a hospital in 30.4% (51/168) of cases and a self-employed private health professional in 13.1% (22/168) of cases.

Furthermore, when we evaluated the perceived effectiveness (on the basis of statement of cure) of each therapeutic option, it appeared that 53.8% of patients felt cured at the time of the survey. Of these, 65.2% (88/135) reported this after having used the initial therapeutic option; 28.1% (38/135) and 6.7% (9/135), respectively, reported feeling cured after the second and the third options. The respective cure rates for the initial therapeutic option were 42.9% (3/7) for traditional medicine, 51.4% (36/70) for self-medication, 71.4% (5/7) for a self-employed private health professional operating outside a health facility, 82.8% (24/29) for FLHS, and 90.9% (20/22) for a hospital.

### Factors influencing the choice of the therapeutic option: bivariate and logistic regression analyses

Table 4 shows the results of the bivariate analysis and indicates that only the chronicity of disease was significantly associated with the choice of formal health care (p = 0.007). When variables were introduced into a logistic regression model, “chronicity of disease” remained the only variable that determined the choice formal health care (adjusted OR = 2.44, 95% CI = 1.24 to 4.78, p = 0.01). This means that patients used formal health care as one of the options for diseases reported as chronic 2.44 times more often than for diseases reported as acute.

**Table 4:** Factors influencing the use of formal therapeutic options in Lubumbashi, 2010

**Factors**	**n = 251**	**Formal therapeutic options (%)**	**OR**	**95%CI**	**p**
Age (years)					0.91
≤ 4	61	41.0	1.00		
≥ 5	190	40.5	0.96	0.49 – 1.88	
Sex					0.47
Male	105	39.1	1.00		
Female	146	41.8	1.16	0.77 – 1.76	
Acute/chronic disease ^a^					0.01
Acute	217	37.3	1.00		
Chronic	34	61.8	2.52	1.25 – 5.09	
Health problems					0.35
Malaria syndrome	86	36.0	1.00		
Respiratory tract syndrome	70	34.3	0.94	0.46 – 1.89	
WHS complex	43	46.5	1.41	0.65 – 3.07	
Others	52	51.9	1.75	0.84 – 3.64	
Household size					0.44
1-5	74	46.0	1.00		
6-10	129	35.7	0.67	0.37 – 1.24	
≥ 11	48	45.8	0.97	0.55 – 1.69	
Number of parents in the household					0.63
1	6	50.0	1.00		
2	245	40.4	0.67	0.13 – 3.37	
Level of education of household head					0.23
Higher education	55	32.7	1.00		
High education	145	40.7	1.34	0,67 - 2,69	
Illiterate and Primary education	51	49.0	1.78	0.83 – 3.81	
Main income generating activity of the household head					0.56
Corporate executives and professionals	6	33.3	1.00		
Salaried employees and independent workers	139	44.6	1.61	0.28 – 9.15	
Paid on a daily basis	77	36.4	1.14	0.19 – 6.71	
Inactive	29	34.5	1.05	0.16 – 6.96	
Socioeconomic status of the household					0.18
5 (Richest)	48	41.7	1.00		
4	52	40.4	0.80	0.38 – 1.73	
3	39	33.3	0.74	0.33 - 1.67	
2	60	43.3	1.41	0.82 - 2.42	
1 (Poorest)	52	42.3	0.75	0.40 – 1.40	

^a^As reported by interviewee.

### Analysis of the direct financial costs associated with the use of various therapeutic options

Table 5 presents the expenses related to each single therapeutic option, according to different components of care. It does not capture the totality of costs because certain episodes of disease were still ongoing at the time of the survey. Drugs were the only expenditure in case of use of the informal health care system and they were the greatest expense when patients used the formal system. Costs were significantly higher in FLHS and in hospitals than in the case of patients that employed self-medication or used traditional medicine. Within the formal system, FLHS expenditures were lower than hospital expenditures; however, the observed differences were not statistically significant with the exception of transportation fees to an FLHS, which were nearly zero.

**Table 5:** **Median costs (in USD**^**a**^**) for the components of care for each therapeutic option, Lubumbashi 2010 (n = 251 households)**

**Therapeutic option/components of care**	**n**	**Median cost (Q1-Q3)**	**95% CI**
Traditional medicine	14	0.4 (0.0 -3.9)	0.0-2.0
Self-medication	111	0.9 (0.2 -2.2)	0.6-1.2
Self-employed private health professionals			
Transportation fees	23	0.0 (0.0-0.0)	0.0-0.0
Consultation fees	21	0.0 (0.0-0.2)	0.0-0.1
Cost of medicines	19	3.4 (1.4-7.8)	1.1-5.7
FLHS			
Transportation fees	100	0.0 (0.0-0.0)	0.0-0.0
Consultation fees	68	2.2 (1.2-2.8)	2.2-2.3
Lab exams fees	67	2.2 (0.0-3.9)	1.5-3.0
Cost of medicines	56	5.9 (1.9-13.6)	3.5-8.4
Cost of observation	78	0.0 (0.0-0.0)	0.0-0.0
Hospital			
Transportation fees	50	1.4 (0.0-3.6)	0.6-2.2
Consultation fees	32	3.3 (0.0-5.2)	1.9-4.8
Lab exams fees	26	1.1 (0.0-5.6)	0.0-2.8
Cost of medicines	25	7.8 (2.2-16.7)	3.2-12.3
Hospitalization fees	35	0.0 (0.0-0.0)	0.0-0.0

^a^1USD = 900 Congolese Francs.

Table 6 indicates that none of the variables studied significantly influenced the direct financial cost of care during an episode of disease. However, two trends must be noted. First, the cost of care was relatively high for patients with diseases in the categories of respiratory tract syndrome and diseases related to the WHS complex. Second, the poorest pay the same as the more wealthy patients for health care.

**Table 6:** **Factors influencing the total direct financial cost (in USD**^**a**^**) in the management of an episode of illness, Lubumbashi 2010**

**Variables**	**n = 95**^**b**^	**Median cost (Q1-Q3)**	**95% CI**
Age			
≤ 4	18	2.6 (1.0-11.5)	0.0-6.5
≥ 5	77	8.9 (2.5-18.3)	6.0-11.7
Sex			
Male	39	8.7 (1.7-21.5)	3.6-13.7
Female	56	6.5 (2.4-14.3)	4.0-9.0
First therapeutic option			
Informal	57	5.7 (1.3-13.5)	2.5-8.8
Formal	38	8.9 (1.8-19.4)	5.2-12.6
Number of therapeutic options used			
1	58	3.3 (1.7-13.3)	0.0-11.6
2	32	7.4 (1.7-23.4)	2.3-12.6
3	5	8.8 (2.5-15.0)	6.2-11.4
Health problems			
Malaria syndrome	31	3.9 (1.0-15.8)	1.1-6.7
Respiratory tract syndrome	23	8.9 (2.7-13.7)	6.0-11.8
WHS complex	15	10.7 (3.5-26.9)	3.4-17.9
Others	26	7.5 (2.0-14.7)	4.8-10.2
Level of education of household head			
Illiterate and primary education	22	11.3 (2.6-18.8)	5.8-16.7
Secondary education	48	5.3 (1.1-17.1)	1.6-8.9
Higher education	25	9.8 (3.3-15.6)	5.9-13.6
Socioeconomic status of the household			
1 (Poorest)	20	8.8 (4.6-15.4)	5.0-12.6
2	23	9.4 (1.7-24.6)	1.9-17.0
3	17	6.1 (1.7-36.1)	0.0-19.3
4	20	8.2 (2.6-15.1)	3.8-12.7
5 (Richest)	15	4.9 (1.7-11.6)	0.9-8.9

^a^1USD = 900 Congolese Francs.

^b^This figure represents the number of surveyed patients whose disease episode was finished at the time of survey, and who indicated the total amount spent during the episode.

## Discussion

As in other similar studies [1,10], the merit of this work lies in its multi-stage sampling frame, which renders the findings representative of the population studied (33 HAs with at least seven households per HA), and rigor in the conduct of the survey. In addition, this survey offers a detailed and innovative description of the therapeutic itineraries of patients in an urban health care system.

However, two limitations of this study have to be pointed out. Firstly, the recruitment of interviewers among modern health professionals (i.e. nurses) who presented themselves as such to the interviewees may have led respondents to under-report the use of traditional medicine. Further investigation using appropriate techniques is needed to accurately estimate the importance of traditional medicine in Lubumbashi. Secondly, we did not examine the severity character of reported diseases, a variable which could have contributed to explain the choice of care.

### Reported health problems

The profile of health problems reported in this study is in agreement with what has been described elsewhere in urban populations in developing countries [30]. Malaria and fevers of unknown origin and diseases of the respiratory and digestive tracts predominate [1,2,30]. In this study, one-third of all surveyed patients had suffered from malaria and/or fever during the study period. Although malaria syndrome is a commonly reported health problem, its frequency in our study is half of the one reported by Kakoma [18]. This difference may be explained by seasonal variations in disease incidence: our study took place in the dry season (July), a period of low transmission of malaria, while the Kakoma study was conducted during the rainy season (March-April), a period of high transmission. Our study period may also explain the high frequency of respiratory tract syndrome, which was dominated by acute respiratory infections. The dry season in Lubumbashi is characterized by cold and dust, two factors that favour the development of these infections.

### Therapeutic options and patients’ itineraries

The health-seeking behaviour of patients in Lubumbashi is complex and involves both formal and informal care. Figure 2 indicates that patients used five different therapeutic options, either exclusively or in combination (i.e., sequentially), during the two weeks preceding the survey.

Self-medication with modern drugs is the initial therapeutic option chosen by the majority of inhabitants of Lubumbashi (54.6%) when they face a health problem. This result is comparable to the respective frequencies of 55.6% and 58.5% observed in Ouagadougou [1] and Cotonou [10]. This practice is widespread in both rural [24,31-33] and urban areas [1,2,34]. The main reason for this practice is its affordability; it involves only the cost of the drugs that are purchased; the patient is thus exempt from other costs related to transportation, consultation of health workers, and various technical examinations [35]. Another reason for the high level of self-medication in the specific context of Lubumbashi may be the intrusive marketing of all sort of drugs via wall posters. Such posters are present in almost every single waiting room of a health worker, and are associated with easy access to drugs without prescription by a professional. In this context, self-medication is more likely to have medical (harmful to the patient) as well as economic consequences (spending money on sometimes non useful drugs). Therefore, the importance of self-medication deserves special consideration, especially because this study also reveals that over one-half of those surveyed consider it to be effective. From a public policy point of view, it appears more appropriate to try to rationalize the practice than to attempt (often in vain) to prohibit it. In this context, one option would be to focus regulatory efforts on a limited list of drugs whose contraindications and risks are significant [5] and develop programs to promote self-medication practices for mild health problems. Experiments of this type of practice in the DR Congo have already been documented in the two rural HDs of Kasongo and Bwamanda [36].

The second most frequent initial therapeutic option taken by patients in Lubumbashi is the search for care in formal health facilities. Other similar studies in other cities of sub-Saharan Africa have made the same observation [1,10,34]. In the present study, it was established that more than one-half of patients (56.5%) who had a contact with a formal health care service as the first step in their health-seeking behaviour did so at the level of a FLHS. The FLHS is supposed to be the privileged entry point into the formal health care system in the DR Congo, and elsewhere [37]. Nevertheless, the proportion of patients who bypass the FLHS is quite high: 30.4% of patients who have used formal care as a first, second, or third option went directly to the hospital. Direct access to the hospital was employed as the first option for 11.9% of patients in Lubumbashi. However, in two studies comparable to ours, only 2.1% of patients in Ouagadougou [1] and 4.4% of patients in Cotonou [10] used the hospital as a first option. This difference is striking. In the urban environment of Lubumbashi, where geographical accessibility is not an issue [14], bypassing the FLHS usually reflects the open competition that exists between the two levels of care [14,38,39]. The reasons for the patients’ attraction to the hospital in the context of Lubumbashi are not yet clear and should be investigated further in order to effectively reorganize health care in this city, especially given the perceived high effectiveness of formal care.

In our study, 7.2% of patients used traditional medicine at some point in their complex therapeutic itinerary. This is slightly lower than the 8.5% observed by Kakoma [18] 10 years ago in the same city. The use of traditional medicine was the first step in the therapeutic itinerary for 4.8% of patients. In two comparable surveys conducted in Ouagadougou and Cotonou more than 10 years ago, the rates of use of traditional medicine as a first option were, respectively, 2.4% and 0.4% [1,10]. According to another survey conducted in Cotonou, nearly 30% of hospitalized patients had used a traditional remedy before hospitalization [24]. The WHO [40] and UNAIDS [41] estimate that 80-85% of the rural and urban sub-Saharan population use traditional medicine. The increasing dominance of modern medicine is one possible explanation for the low use of traditional medicine, at least as a first option, observed in our study. However, an examination of patients’ therapeutic itineraries indicates that formal care is the last resort of patients seeking care in the city of Lubumbashi.

### Determinants of therapeutic itineraries in Lubumbashi

Contrary to our expectations (Figure 1), no statistically significant association was observed between the dependent variable (use of formal care) and most of the independent variables (Table 4). Other studies have shown that all of these variables can influence health-seeking behaviour [3,42-45], even if the influence of some of these variables on the choice of therapeutic options is divergent. A study in Guinea-Conakry has shown that children and women preferentially seek care in public health centres, while men rather attend private pharmacies [46]. However, similarly to our study, other studies have shown that sex, household size, education level, and occupation of the head of household do not influence the choice of care [1,8]. Studies also diverge on the recognition of socioeconomic status as a determinant of therapeutic choice. Although some report that high socioeconomic status is a major determinant of the use of modern health care [1,45,47], our findings do not confirm this.

Our study indicates that the nature of a health problem does not determine patients’ therapeutic choices. But, if patients acknowledge that they have a chronic disease, they rather seek formal care than for an acute disease. The validity of this finding is confirmed when looking at the result of cross check with the duration of disease. This further strengthens the finding that formal care is the ultimate recourse for patients in search for health.

### Cost of care

As reported in other studies [35,48,49], our research points to the importance of drugs in health expenditure. Unsurprisingly, using FLHS and hospitals leads to a higher expenditure for drugs than does self-medication. The lack of rationalization of drug prescription, which is exemplified by the over-prescription of antibiotics with regular prescription of more than four drugs, and the prescription of specialty drugs rather than generics, contributes to the high expenditure for drugs [50,51]. Indeed, Kasongo [51] showed that cefotaxime, a third-generation cephalosporin antibiotic, is the antibiotic most often prescribed in two major hospitals in Lubumbashi. Although our study did not investigate the details of the practice of self-medication as it occurs in Lubumbashi, we hypothesize that self-medication may be inadequate because cost of drug creates a barrier that prevents people from taking them at the appropriate dosage and for a sufficiently long period of time.

The results of our study clearly indicate that the total financial cost of an episode of disease is not significantly influenced by any of the analyzed variables. However, a number of trends with respect to health care expenditure deserve to be commented. The relatively high cost of care regarding diseases related to the respiratory tract syndrome and to the WHS complex can only be explained by the high cost of drugs, including the lack of generic antibiotics [50,51]. Surprisingly, the cost of care does not differ statistically between sub-populations of different socio-economic status, and the poorest pay the same as the more wealthy patients. This finding clearly points to the regressive nature of the health care payments in Lubumbashi, which leads to greater impoverishment of the poorest people, as has been reported by other authors [49,52,53].

## Conclusions

Self-medication as a widely spread practice must be rationalized. This process must be part of a more comprehensive drug policy program that should promote the effective use of generic and essential drugs of sound quality. Although formal care is not the most widely used initial therapeutic option, it is the last resort for most patients who appreciate its effectiveness, especially when they believe having a chronic disease. Therefore, decision makers should improve its access to the general population. Current therapeutic itineraries of patients in the health care system of the city of Lubumbashi are complex, but still favourable to the organization of an efficient referral system to ensure continuity of care and complementarities between different levels of care (individual/community, FLHS, hospital). Finally, the highly regressive nature of the current payment system of care calls for dramatic reform to promote mechanisms of universal access to care to ensure solidarity and equity. Our study provides evidences for such actions.

## Competing interests

The authors declare that they have no competing interests.

## Authors’ contributions

MFC, JVDV, VV, and BC conceptualized the study and designed the questionnaire. MFC supervised the data collection and did data validation. VV performed the statistical analysis. MFC drafted the manuscript. JVDV, NOL, MAM, and BC reviewed critically the manuscript. BC supervised the overall research. All authors read and approved the final manuscript.

## Pre-publication history

The pre-publication history for this paper can be accessed here:

http://www.biomedcentral.com/1472-6963/14/173/prepub
